# LINC01128 regulates the development of osteosarcoma by sponging miR‐299‐3p to mediate MMP2 expression and activating Wnt/β‐catenin signalling pathway

**DOI:** 10.1111/jcmm.16046

**Published:** 2020-10-27

**Authors:** Qiang Yao, Ting Chen

**Affiliations:** ^1^ Department of Orthopedics Shengjing Hospital of China Medical University Shenyang China

**Keywords:** bone tumour, ceRNA, invasion, lncRNA, proliferation

## Abstract

Osteosarcoma (OS) is one of the most common metastatic bone cancers, which results in significant morbidity and mortality. The important role of long non‐coding RNAs (lncRNAs) in the biological processes of OS has been demonstrated through several studies. In the current study, we evaluated the role of the lncRNA, LINC01128, in OS. We analysed the expression of LINC01128 in three OS gene expression omnibus (GEO) data sets GSE21257, GSE36001 and GSE42352. The expression of LINC01128 in OS tissues and matched non‐tumour tissues obtained from 50 OS patients was detected using qRT‐PCR. The association between LINC01128 expression and overall survival of OS patients was evaluated using the Kaplan‐Meier method. The effects of LINC01128 knockdown and overexpression were evaluated through in vitro and in vivo assays. The LINC01128/miR‐299‐3p/ MMP2 axis was verified using dual‐luciferase reporter assay and qRT‐PCR assays. GEO data sets analysis revealed that the expression of LINC01128 was increased in OS. Elevated LINC01128 expression was accompanied by shorter overall survival in OS patients. Functional studies revealed that LINC01128 knockdown reduced the proliferation, migration and invasion of OS cells both in vitro and in vivo. Mechanistically, LINC01128 sponged miR‐299‐3p to increase MMP2 expression. Rescue assays determined the role of the LINC01128/miR‐299‐3p/MMP2 axis in the proliferation, migration and invasion of OS cells. Additionally, the Wnt/β‐catenin signalling pathway was activated by LINC01128 and MMP2 in OS cell lines. In summary, this study demonstrates that LINC01128 facilitates OS by functioning as a sponge of miR‐299‐3p, thus promoting MMP2 expression and activating the Wnt/β‐catenin signalling pathway.

## 1 INTRODUCTION

Osteosarcoma (OS) is the most frequent primary malignant bone tumour and occurs primarily in children and young adolescents.^1^, ^2^ It is characterized by mesenchymal cells, with a high degree of genetic heterogeneity, that produce immature bone and osteoid.^3^, ^4^ In the 1970s, amputation was the only intervention for OS patients, and the 5‐year survival rate was <20%. The development of chemotherapy has significantly improved life expectancy, and the 5‐year survival rate is currently 65%‐70%.^5^, ^6^, ^7^ Nevertheless, with the changes in the management strategies, the overall survival rate for ten years has stabilized since the 1990s.^8^, ^9^, ^10^ Moreover, no breakthrough has been made in the fields of clinical and scientific research of OS. Therefore, a better understanding of the biological processes and pivotal mechanisms promoting OS tumorigenesis and development are extremely important to improve the efficacy of treatments and prognosis in OS patients.

Long non‐coding RNAs (lncRNAs) are a type of RNA with lengths exceeding 200 nucleotides that do not encode proteins.^11^ LncRNAs have attracted increasing attention as cancer biomarkers for early screening, diagnosis, prognosis and response to drug treatment.^12^, ^13^, ^14^ Although several lncRNAs including lncRNA ODRUL,^11^, ^15^ SNHG15,^16^ SNHG16,^17^ SNHG4^18^ and AFAP1‐AS^19^ have been confirmed to be highly correlated with OS, no widely applicable therapeutic targets have not been identified thus far. *LINC01128* has been reported to be associated with acute myeloid leukaemia^20^ and cervical cancer.^21^ However, whether *LINC01128* is involved in the pathophysiology of OS remains unknown.

In this study, we screened *LINC01128* as a potential OS target through the analyses of three GEO databases and evaluated its expression in tissues from OS patients and cell lines. We performed functional studies and investigated its molecular mechanism in OS.

## 2 MATERIAL AND METHODS

### 2.1 Collection of samples

OS tissue samples and matched adjacent non‐tumour tissues (fifty pairs) were collected from patients who underwent surgery at our hospital. Informed consents were obtained from all the patients or their guardians. None of the patients received chemotherapy prior to the surgery. Tissue samples were collected during definitive surgery and stored in liquid nitrogen until use. All patients were diagnosed with OS based on histopathological examination.

### 2.2 Cell culture

The cell bank of the Chinese Academy of Sciences (Shanghai, China) provided the OS cell lines (Saos2, MG63, U2OS and HOS) and a human normal osteoblast cell line hFOB1.19. MG63, HOS and U2OS cells were cultured in alpha minimal essential medium (α‐MEM; HyClone, Logan, UT, USA) supplemented with 10% foetal bovine serum (FBS; Gibco, Carlsbad, CA, USA). Saos2 cells were cultured in Rosewell Park Memorial Institute 1640 (RPMI‐1640) medium (Gibco, USA) supplemented with 15% FBS. hFOB1.19 cells were cultured in Ham's F12/Dulbecco's modified Eagle's medium (DMEM, Gibco) supplemented with 10% FBS, 100 U/mL penicillin and 100 mg/mL streptomycin (Invitrogen, Grand Island, NY, USA). All four human OS cell lines were maintained in a humidified incubator at 37°C with a 5% CO_2_. The hFOB 1.19 cell line was maintained at 33.5°C with a 5% CO_2_.

### 2.3 RNA transfection

ShRNAs against *LINC01128*, matching negative control (sh‐NC), shRNAs against *MMP2* (sh‐*MMP2*) and its matching negative control (sh‐NC) were commercially acquired and synthesized by Ribo Bio Corporation (Guangzhou, China). For the knockdown of miR‐299‐3p, miR‐299‐3p mimics, miR‐299‐3p inhibitor and their corresponding NCs (inhibitor‐NC and miR‐NC) were synthesized by Ribo Bio Corporation.

Lentiviral infection was used to generate various stably transfected cell lines. Briefly, 293T cells were co‐transfected with packaging plasmids as well as viral vectors. After 48 hours of transfection, the culture medium was supplemented with 5 μg/mL polybrene, filtered through a 0.45 μm filter and used to infect the target cells. After 36 hours of infection, the cells were selected in culture medium containing 2 μg/mL puromycin.

### 2.4 Extraction of RNA and qRT‐PCR analysis

Total RNA was isolated from the OS tissues or cultured cells using TRIzol^®^ reagent (Invitrogen, Carlsbad, CA, USA). The RNA was reverse transcribed into cDNAs using Transcriptor First Strand cDNA Synthesis kit (Roche Diagnostics, Pleasanton, CA, USA) at 37°C for 15 min and 85°C for 5 s following the manufacturer's instructions. qPCR was performed using the SYBR Green PCR kit (Applied Biosystems, Foster City, CA, USA) by initial denaturation at 94°C for 5 minutes, followed by 40 cycles including denaturation at 94°C for 30 seconds, annealing at 55°C for 30 seconds and extension at 72°C for 90 seconds. The relative expression level was calculated using the 2^−ΔΔCt^ method and glyceraldehyde 3‐phosphate dehydrogenase (GAPDH) or U6 was used as an endogenous reference gene. Primers used in the PCR are listed in Table S1.

### 2.5 Counting Kit‐8 (CCK‐8) assay

OS cell proliferation was determined using the CCK‐8 assay. Specifically, U2OS and Saos2 cells were plated at 2 × 10^3^ cells/well in 96‐well plates (containing complete growth medium) and incubated in a moist environment with 5% CO_2_ at 37°C for 12 hours CCK‐8 reagent (10 μL/well; Dojindo, Japan) was added on days 1, 2, 3 and 4 followed by continuous incubation at 37°C for 2 hours and a microplate reader (Bio‐Rad, Hercules, CA, USA) was used to read the absorbance at 450 nm.

### 2.6 5‐ethynyl‐2’‐deoxyuridine (EdU) incorporation assay

Saos2 and U2OS cells were plated at 8 × 10^3^ cells/well in a 96‐well plate and incubated overnight, followed by transfection for 48 hours. Subsequently, EdU solution (25 μmol/L) was added to the cell culture medium and the cells were incubated for 4 h. After fixing in 4% paraformaldehyde for 30 minutes and permeabilizing with 0.5% Triton X‐100 (Sigma, St. Louis, MO, USA) for 10 minutes, the cell nuclei were stained using DAPI (5 μg/mL) for 10 minutes. The images were collected using a fluorescent microscope (Leica, Wetzlar, Germany), and the EdU‐positive cells were quantified in five randomly selected fields.

### 2.7 Colony formation assay

U2OS or Saos2 cells (~1 × 10^3^ cells/well) were plated in 6‐well plates. Cells were fixed with methanol and stained with 0.1% crystal violet after 14 days. Colonies containing over 50 cells in size were counted manually. The experiments were repeated thrice to obtain the average colony formation rate.

### 2.8 Wound healing assay

Cells were cultured in 6‐well plates. When the cells reached 90% confluence, the cell layers were scratched using a 20 μL tip to form wounded gaps, gently washed to remove displaced cells and cultured for 24 hours. The wounded gaps were photographed at different time points with the same microscope setting, and the gap width covered by the migrating cells in different areas of each wound was measured and analysed.

### 2.9 Invasion assay

Invasion assay was performed using Transwell pre‐coated Matrigel chambers according to the manufacturer's protocol (BD Science, Bedford, MA, USA). For cell invasion assay, 800 µl medium supplemented with 10% FBS was added to the lower well, and 5 × 10^5^ cells in 100 µL serum‐free medium were seeded into the upper chamber of a Transwell plate. After 24 hours of incubation, 10‐min cell fixation was performed in 4% polymethanol, followed by 20 minutes staining using 0.1% crystal violet (Beyotime, Nantong, China). The invasion rate was quantified by counting the invasive cells in at least three random fields.

### 2.10 In vivo assay

Male BALB/c nude mice (6‐week‐old) were purchased from Vital River Laboratories Company (Beijing, China) and maintained in special pathogen‐free (SPF) condition. The animal experiments were carried out in strict accordance with the recommendations in the Guide for the Care and Use of Laboratory Animals of the National Institutes of Health. The mice were cultured under standard conditions (24°C ± 2°C, 50 ± 10% relative humidity, 12‐hours light/dark cycles) and with unlimited access to standard rodent maintenance feed (KEAO XIELI FEED Co., Ltd., Beijing, China) and water. Hygienic conditions were maintained by weekly cage changes. Animal health and behaviour were monitored every day, and body weights were assessed weekly over the course of the study. Mice were injected with 5 × 10^6^ Saos2 cells that stably express sh‐NC or sh‐*LINC01128*, subcutaneously in both flanks. Tumours were measured once a week with a calliper and the tumour volume (mm^3^) was calculated as 0.5 × Length × Width^2^. All animals were killed by overdose (>120 mg/kg body weight) intraperitoneal injection of pentobarbital after 28 days. The death was verified by loss of spontaneous breathing. The xenograft tumour tissues were sampled for subsequent analyses.

### 2.11 Nuclear and cytoplasmic fractionation

Nuclear and cytoplasmic fractionation assay was performed using the PARIS™ kit (Invitrogen) following the manufacturer's instructions. Cell fractionation solution was used to lyse the Saos2 and U2OS cells, followed by centrifugation to isolate the cytoplasmic fractions. Next, the remaining cytoplasmic fractions were discarded and the cell supernatant was placed into cell disruption buffer and incubated on ice. The cell supernatant and lysate were suspended in a mixture consisting of equal volume of 2 × lysis/binding and ethanol. Finally, the nuclear and cytoplasmic RNAs were eluted and extracted using TRIzol reagent (Life Technologies, Carlsbad, CA, USA). GAPDH was used as the cytoplasmic control, while U6 as the nuclear control.

### 2.12 Luciferase reporter assay

Wild‐type (Wt) and mutant (Mut) fluorescent plasmids corresponding to *LINC01128* and *MMP2* (*LINC01128*‐Wt, *MMP2*‐Wt, *LINC01128*‐Mut and *MMP2*‐Mut) were obtained from Promega Corporation (Fitchburg, Wisconsin, USA). Following plating in 96‐well plates, 293T cells were co‐transfected with miR‐299‐3p mimics or miR‐NC. Renilla luciferase activity was used for normalization of the luciferase activity. A dual‐luciferase reporter assay system (Promega) was used for the luciferase reporter assay.

### 2.13 Western blotting assay

The concentration of protein was determined using the BCA Protein Assay kit (Beyotime, Nantong, China). The samples (40 µg protein per lane) were separated by sodium dodecyl sulphate polyacrylamide gel electrophoresis (SDS‐PAGE, 10% gel) and transferred to a Hybond membrane (Amersham, Germany). The membrane was blocked with 5% skimmed milk for 2 hours, followed by incubation with *MMP2* (ab97779) and c‐Myc (ab39688), β‐catenin (ab16051), GAPDH (ab181602) and cyclin D1 (ab227561) primary antibodies (diluted at 1:1000; Abcam, UK) at 4°C overnight. The membranes were rinsed with Tris‐buffered saline‐Tween (TBST) thrice followed by incubation with secondary antibodies for 2 hours. Enhanced chemiluminescence (ECL) reagent (Santa Cruz Biotechnology, CA, USA) was used to visualize the protein bands in the dark. The protein bands were analysed using ImageJ software (version 1.48; NIH; National Institutes of Health, Bethesda, MD, USA).

### 2.14 Statistical analysis

SPSS 22.0 software (SPSS Inc, Chicago, IL, USA) was used for statistical analyses. The data of all assays (tested individually at least thrice) in this study are expressed as the mean ± standard deviation (SD). The statistically significant differences between the two groups were determined using Mann‐Whitney *U* test, Wilcoxon signed‐rank test or two‐tailed Student's *t* test, where appropriate. The comparisons among different groups (>2 groups) were analysed by one‐way analysis of variance (one‐way ANOVA) with a Tukey's post hoc test. Data concerning the association of *LINC01128* expression with clinicopathological features of OS are analysed by chi‐squared test and Fisher's exact test. Additionally, the log‐rank test and Kaplan‐Meier method were used to evaluate the overall survival. Expression correlations were investigated using Pearson correlation analysis. *P* < .05 was considered statistically significant.

## 3 RESULTS

### 3.1 LINC01128 level is increased in OS and associated with poor prognosis in OS patients

**FIGURE 1:**
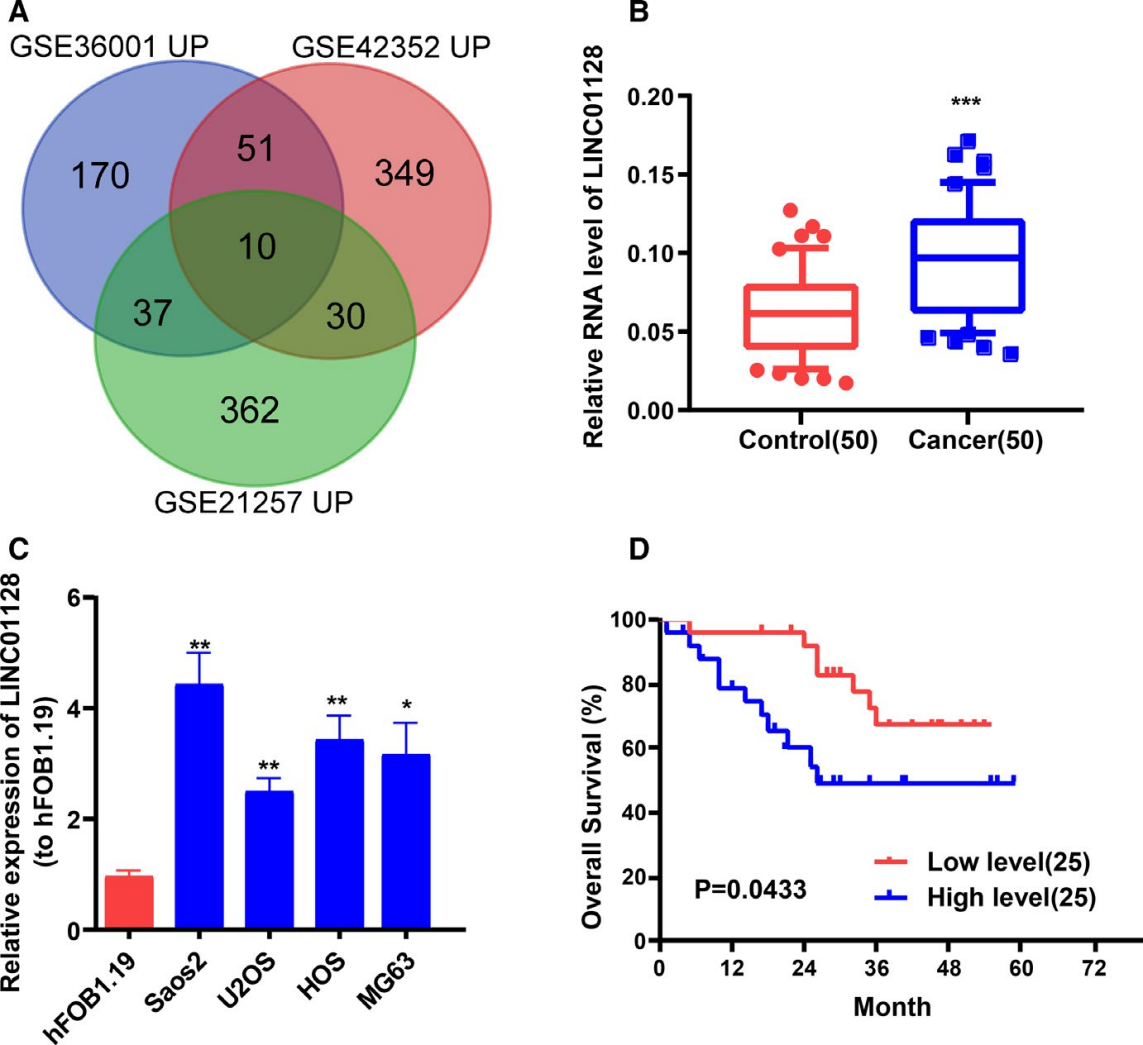
*LINC01128* expression is associated with poor prognosis in OS patients. (A) Three osteosarcoma GEO data sets (GSE21257, GSE36001, GSE42352) were analysed and ten highly expressed genes were identified from the intersection. (B) QRT‐PCR analysis of *LINC01128* expression in 50 pairs of OS and normal tissues obtained from OS patients. (C) *LINC01128* expression in OS cell lines and in hFOB1.19. (D) Kaplan‐Meier curve showing the overall survival of OS patients stratified based on *LINC01128* expression. ^*^ *P* < .05, ^**^ *P* < .01 and ^***^ *P* < .001

First, we analysed three OS GEO data sets GSE21257, GSE36001 and GSE42352 (Figures [Link], [Link], [Link]). After merging and comparing of the data sets, we identified ten highly expressed genes (Figure 1A). *LINC01128* was the only lncRNA retrieved among them. With the objective to study the pathogenesis of OS from an epigenetic perspective, we chose to focus on LINC01128. qRT‐PCR assay for LINC01128 expression in the OS tissues and matched non‐tumour tissues obtained from 50 OS patients demonstrated that OS tissues displayed higher expression of LINC01128 relative to the paired paracancerous tissues (Figure 1B). Similarly, LINC01128 was also found to be expressed at a higher level in the OS cell lines (Saos2, MG63, U2OS and HOS) relative to that in the human normal osteoblast hFOB1.19 cells (Figure 1C). Based on the median expression level of LINC01128 in the 50 pairs of tissue specimens, they were classified into high and low LINC01128 expression groups. The overall survival rate of OS patients in high LINC01128 expression group was significantly lower than that in the low LINC01128 expression group (Figure 1D). The association between *LINC01128* expression and OS clinicopathological features is presented in Table 1. It was discovered that increased *LINC01128* was observably correlated with tumour size, distant metastasis and clinical stage, but other clinical and pathological characteristics such as age and gender were found to have no evident association with *LINC01128* expression.

**TABLE 1:** Association of *LINC01128* expression with clinicopathological features of osteosarcoma

Feathers	Number	High	Low	*P*‐value
All cases	50	25	25	
Age(y)				
<18	26	12	14	.7775
≥18	24	13	11	
Gender				
Male	28	14	14	>.9999
Female	22	11	11	
Tumour size (cm)				
<5	21	6	15	**.0209**
≥5	29	19	10	
Distant metastasis				
Absent	23	17	6	**.0041**
Present	27	8	19	
Clinical stage				
I‐IIA	24	6	18	**.0016**
IIB‐III	26	19	7	

Note

Total data from 50 tumour tissues of osteosarcoma patients were analysed. For the expression of *LINC01128* was assayed by qRT‐PCR, the median expression level was used as the cut‐off. Data were analysed by chi‐squared test and Fisher's exact test. *P*‐value in bold indicates statistically significant.

### 3.2 LINC01128 affects the proliferation, migration and invasion of OS cells

**FIGURE 2:**
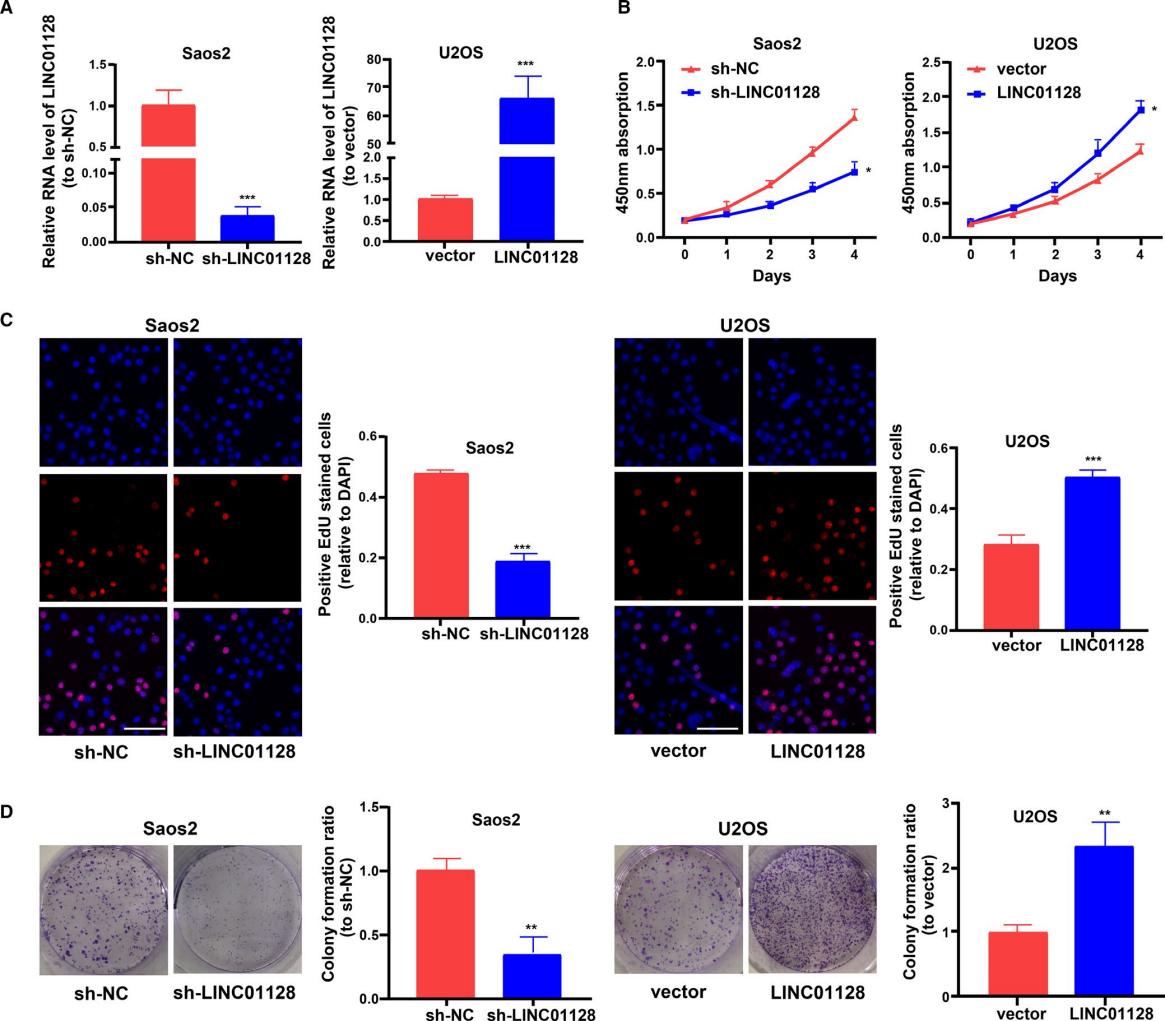
Effect of *LINC01128* on the proliferation of OS cells. (A) Transfection efficiency of sh‐*LINC01128* and *LINC01128* overexpression vector. (B) CCK‐8, (C) EdU (bar = 100 μm) and (D) colony formation assays confirmed the effect of *LINC01128* knockdown and overexpression on the proliferation of OS cells. ^*^ *P* < .05, ^**^ *P* < .01 and ^***^ *P* < .001

**FIGURE 3:**
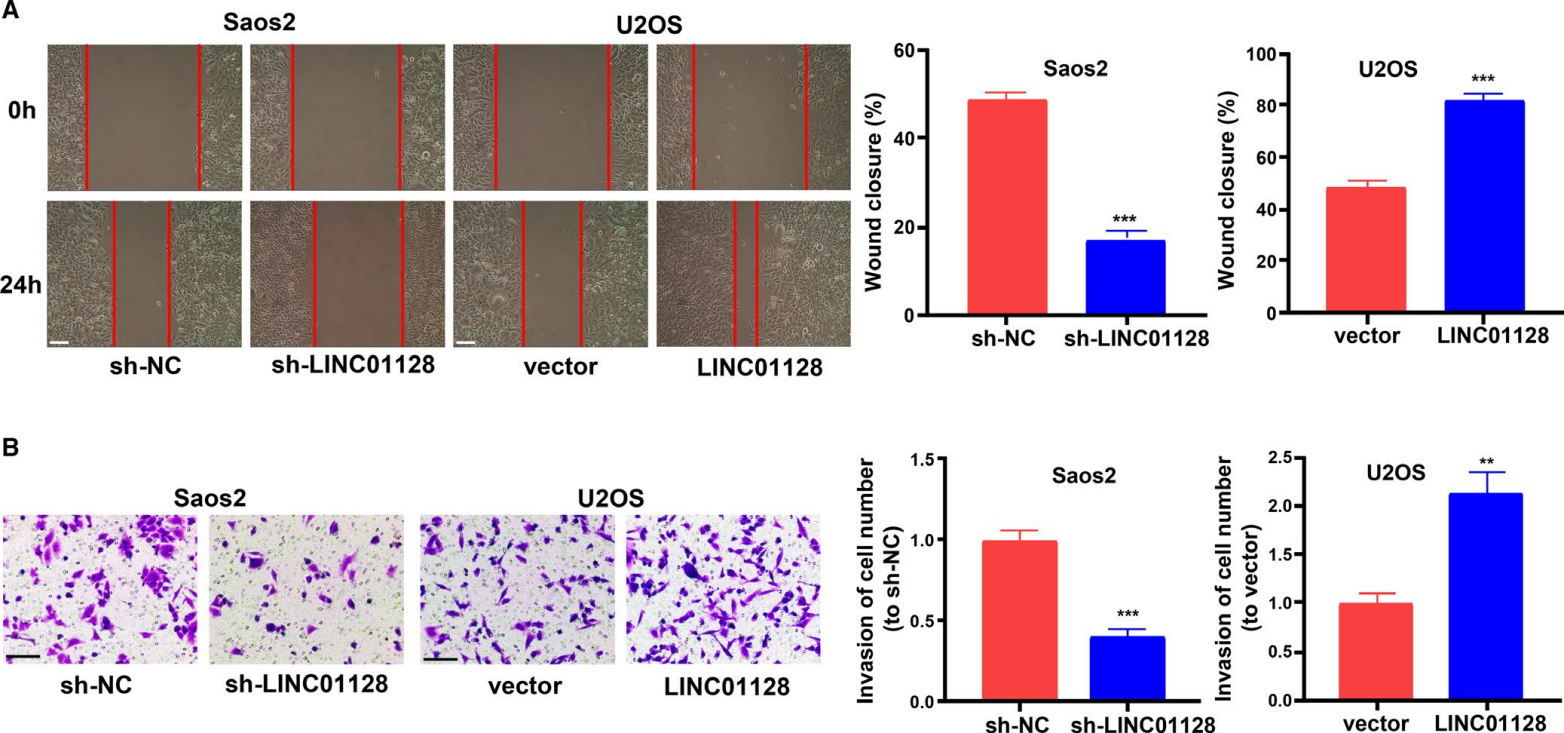
Effect of *LINC01128* on migration and invasion of OS cells. (A) Migration of Saos2 and U2OS cells were analysed using wound healing assay (bar = 100 μm). (B) Invasion of Saos2 and U2OS cells were analysed using Transwell assay (bar = 100 μm). ^**^ *P* < .01 and ^***^ *P* < .001

LINC01128 gain‐ or loss‐of‐function was performed in U2OS and Saos2 cells using LINC01128 overexpression construct or shRNA, respectively. Figure 2A shows the transfection efficiency of sh‐LINC01128 and LINC01128 overexpression in Saos2 and U2OS cells. CCK‐8 and EdU, assays confirmed that sh‐LINC01128 inhibited the proliferation of OS cells, while overexpression of LINC01128 promoted cell proliferation (Figure 2B, C). Interestingly, we also observed similar phenomenon in the hFOB1.19 cells (Figure S5). Colony formation assays further verified these results by showing that the number and size of all cell colonies were enhanced by LINC01128 up‐regulation and inhibited by sh‐LINC01128 compared with the control group (Figure 2D). Additionally, wound healing assay showed that sh‐LINC01128 inhibited the migration of OS cells, while overexpression of LINC01128 promoted cell migration (Figure 3A). Invasion assay also revealed that the invasion of Saos2 cells was inhibited by sh‐LINC01128, and conversely, invasion of U2OS cells was promoted by overexpression of LINC01128 (Figure 3B). These findings indicate that high LINC01128 level stimulates the growth and metastasis of OS cells.

### 3.3 Down‐regulation of LINC01128 inhibits tumour growth and metastasis in vivo

**FIGURE 4:**
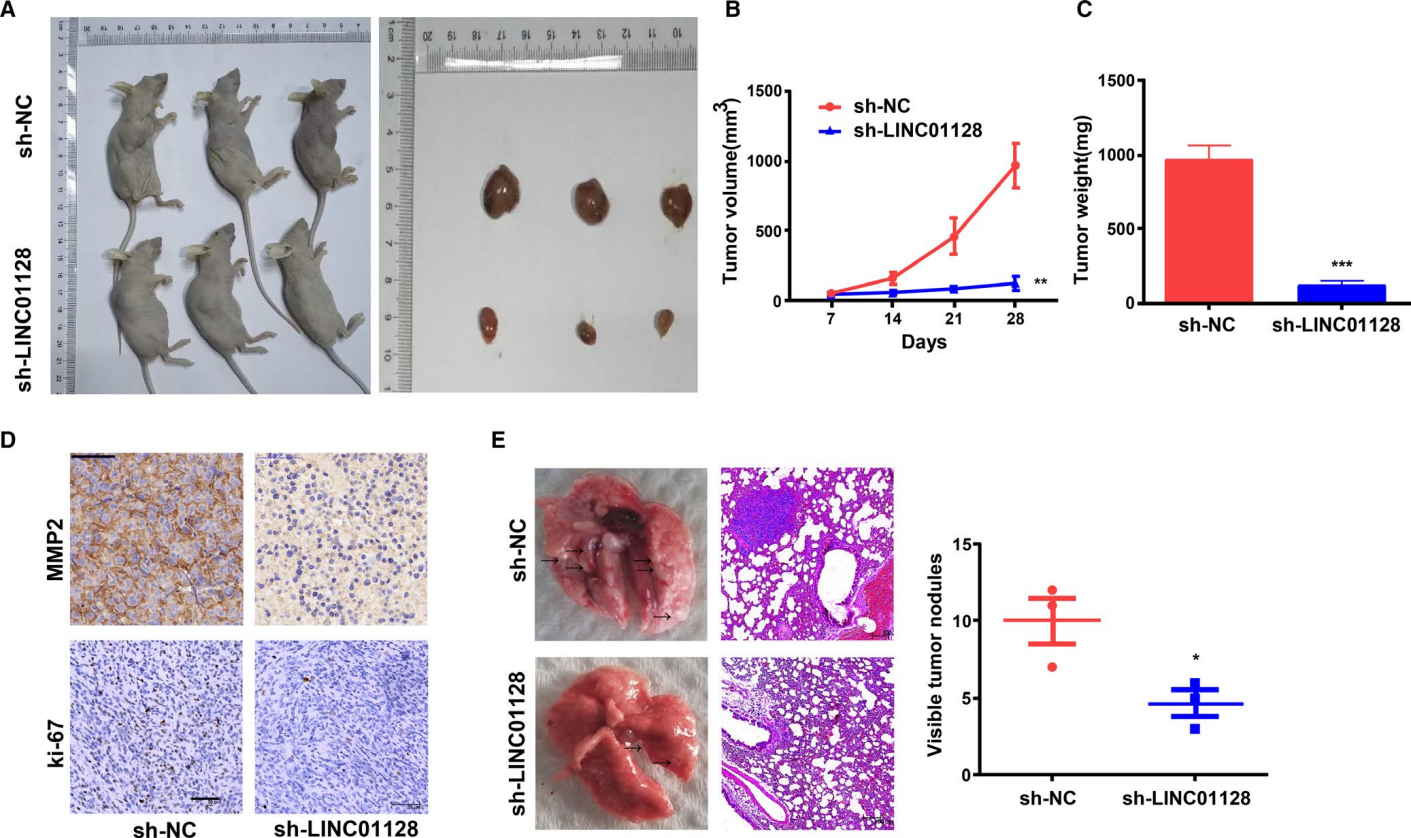
Sh‐*LINC01128* inhibits the OS tumour growth and metastasis. (A) Xenograft OS tumours. (B) Growth of xenograft tumours formed by sh‐*LINC01128* cells is slower than that of xenograft tumours formed by sh‐NC cells. (C) Mean weight of xenograft tumours. (D) Sh‐*LINC01128* markedly reduced *MMP2* and Ki‐67 in tumours compared with negative control (bar = 50 μm). (E) Representative macroscopic and microscopic images (H&E staining) of the lung tissue sections. **P < .01 and ***P < .001

An in vivo xenograft model of OS growth was generated by transplanting Saos2 cells subcutaneously into mice. Using this model, we found that the *LINC01128* knockout group (sh‐*LINC01128*) exhibited significantly less tumour propagation than the sh‐NC group (Figure 4A). The average volume and weight of the xenograft tumours in the sh‐*LINC01128* group were lower than that in the sh‐NC group (Figure 4B and C). Staining of the tumour sections revealed that the expression level of *MMP2* and Ki‐67 was higher in the sh‐NC group compared with the sh‐*LINC01128* group (Figure 4D). To evaluate the potential involvement of *LINC01128* in lung metastasis, we additionally generated a metastatic model and found that the number of lung metastases was clearly lower in the sh‐*LINC01128* group compared with the sh‐NC group (Figure 4E). Together, these findings indicate that *LINC01128* is involved in the propagation and metastasis of OS in vivo.

### 3.4 LINC01128 functions as a molecular sponge of miR‐299‐3p

**FIGURE 5:**
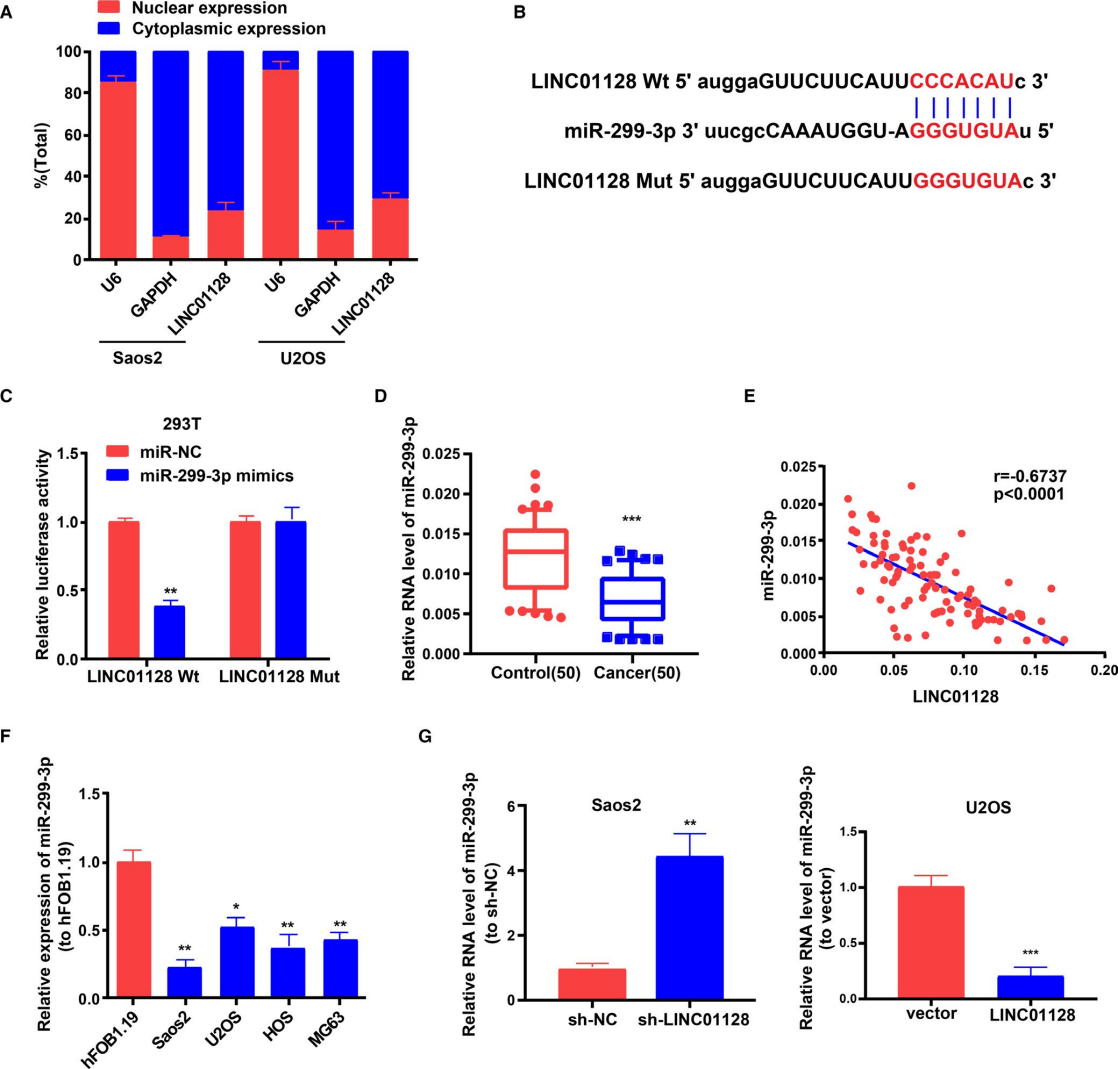
*LINC01128* serves as a molecular sponge of miR‐299‐3p. (A) The localization of *LINC01128* in OS cells was assessed using subcellular fractionation assay. (B) The putative binding sites between *LINC01128* and miR‐299‐3p. (C) Dual‐luciferase reporter assay in 293T cells. (D) MiR‐299‐3p expression in OS tissues. (E) *LINC01128* expression negatively correlates with miR‐299‐3p expression in OS tissues (*r* = −0.6737). (F) MiR‐299‐3p expression in OS cell lines and in hFOB1.19. (G) *LINC01128* negatively regulates the expression of miR‐299‐3p in OS cells. ^**^ *P* < .01 and ^***^ *P* < .001

We examined whether *LINC01128* modulates gene expression through acting as a miRNA sponge. The localization of *LINC01128* in the OS cells was assessed through subcellular fractionation assay and found to be enriched in the cytoplasm in OS cells (Figure 5A). Through StarBase (http://starbase.sysu.edu.cn/) analysis, we found that there are multiple miRNAs interacting with *LINC01128*. According to the level of 'clip ExpNum', we selected the top 20 miRNAs as the potential target miRNAs with the strongest interaction with *LINC01128* (Table S2). Among the 20 potential target miRNAs, miR‐299‐3p is the most obviously down‐regulated miRNA in OS tissues (Figure S4). Thus, miR‐299‐3p was selected as the potential target of *LINC01128*. The putative binding sites shared by *LINC01128* and miR‐299‐3p are shown in Figure 5B. Dual‐luciferase reporter assay in 293T cells revealed that overexpression of miR‐299‐3p reduced the luciferase activity of *LINC01128*‐Wt, but not that of *LINC01128*‐Mut (Figure 5C). Based on qRT‐PCR results, it was demonstrated that miR‐299‐3p expression level in OS tissues was obviously lower than that in the adjacent normal tissues (Figure 5D). Furthermore, miR‐299‐3p expression in the OS tissues was inversely related to *LINC01128* expression (Figure 5E). Similarly, miR‐299‐3p was also found to be expressed at a lower level in the OS cell lines (Saos2, MG63, U2OS and HOS) relative to that in the human normal osteoblast hFOB1.19 cells (Figure 5F). QRT‐PCR validated that *LINC01128* negatively regulated the expression of miR‐299‐3p in OS cells (Figure 5G). These results demonstrate that *LINC01128* functions as a miR‐299‐3p sponge in OS cells.

### 3.5 MMP2 is the target of miR‐299‐3p

**FIGURE 6:**
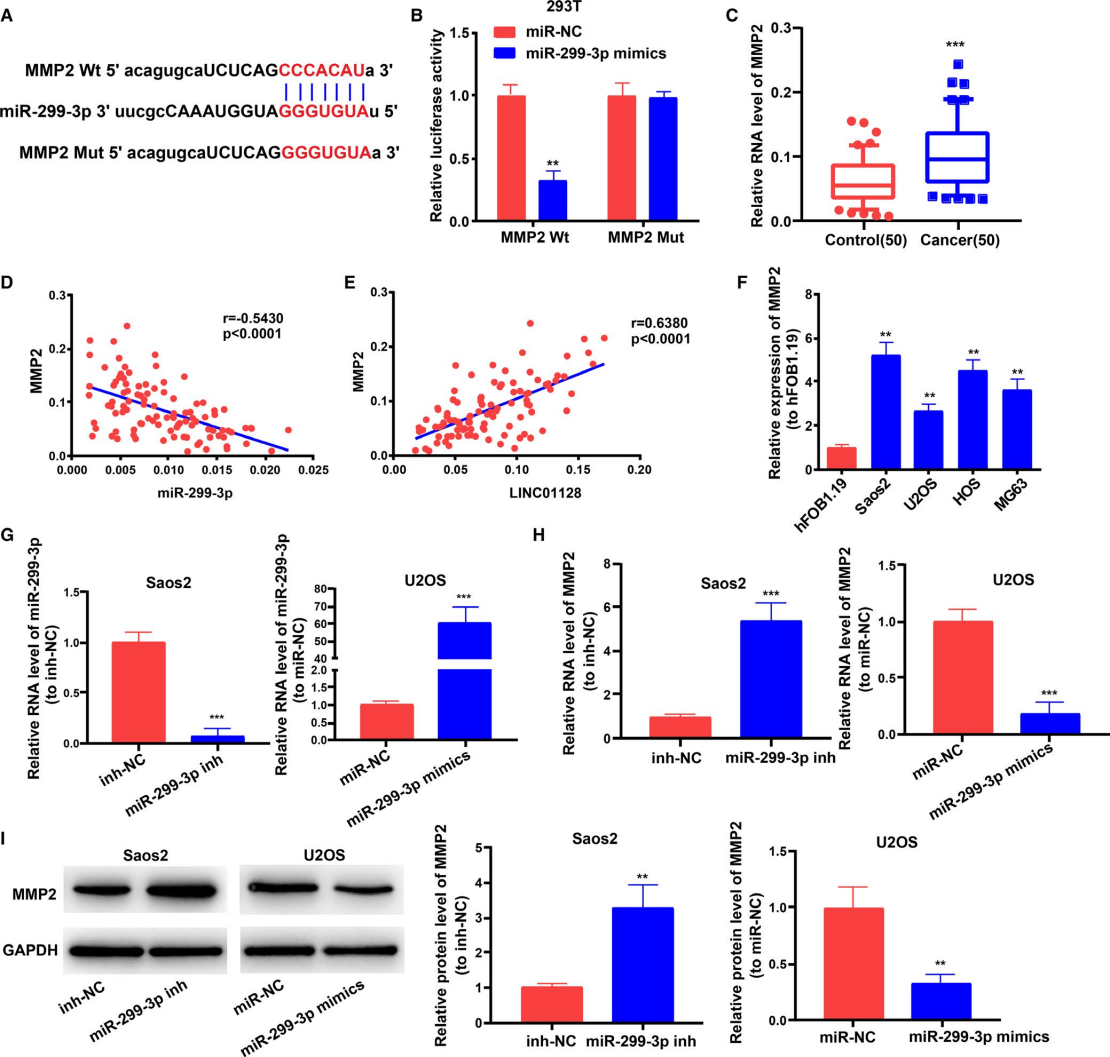
*MMP2* is a target of miR‐299‐3p. (A) The binding sites of miR‐299‐3p in the 3'UTR of *MMP2* were predicted. (B) Dual‐luciferase reporter assay in 293T cells. (C) *MMP2* expression level in OS tissues. (D) Pearson correlation analysis showed that the expression of *MMP2* is inversely correlated with miR‐299‐3p expression in OS tissues (*r* = −0.5430). (E) *MMP2* expression is positively correlated with *LINC01128* expression in OS tissues (*r* = 0.6380). (F) *MMP2* expression in OS cell lines and in hFOB1.19. (G) Transfection efficiency of miR‐299‐3p mimics and miR‐299‐3p inhibitor. (H, I) RNA and protein level of *MMP2* in OS cells after transfection with miR‐299‐3p mimics or inhibitors. ^**^ *P* < .01 and ^***^ *P* < .001

Through StarBase (http://starbase.sysu.edu.cn/) analysis, we found that there are multiple mRNAs interacting with miR‐299‐3p. Through taking the intersection of four databases in StarBase (PITA, miRmap, microT and miRanda), we selected 131 potential target genes. According to the level of 'clip ExpNum', we selected the top 11 mRNAs as the potential target genes with the strongest interaction with miR‐299‐3p (Table S3). Among the 11 potential target genes, only MMP2 is reported to be closely related to the pathogenesis of osteosarcoma.^15^, ^22^ So, we identified *MMP2* as the potential target of miR‐299‐3p, and the potential binding sites of miR‐299‐3p in the 3'UTR of *MMP2* were also identified (Figure 6A). Dual‐luciferase reporter assay showed that miR‐299‐3p mimics clearly reduced the luciferase activity of *MMP2*‐Wt reporter in 293T cells (Figure 6B). Besides, the expression of *MMP2* was increased in the OS tissues (Figure 6C), which is negative correlated with mir‐299‐3p (Figure 6D) but positive correlated with *LINC01128* (Figure 6E). Similarly, *MMP2* was also found to be expressed at a higher level in the OS cell lines (Saos2, MG63, U2OS and HOS) relative to that in the human normal osteoblast hFOB1.19 cells (Figure 6F). To further verify the relationship between miR‐299‐3p and *MMP2*, we transfected miR‐299‐3p mimics in U2OS cells and used a miR‐299‐3p inhibitor in Saos2 cells (Figure 6G) and then examined *MMP2* expression level using qRT‐PCR and Western blotting. Both the RNA and protein levels of *MMP2* were inversely regulated by miR‐299‐3p (Figure 6H and I). These data demonstrate that *MMP2* is a target of miR‐299‐3p in OS cells.

### 3.6 LINC01128 affects the proliferation and invasion of OS cells through collaborating with miR‐299‐3p and MMP2

**FIGURE 7:**
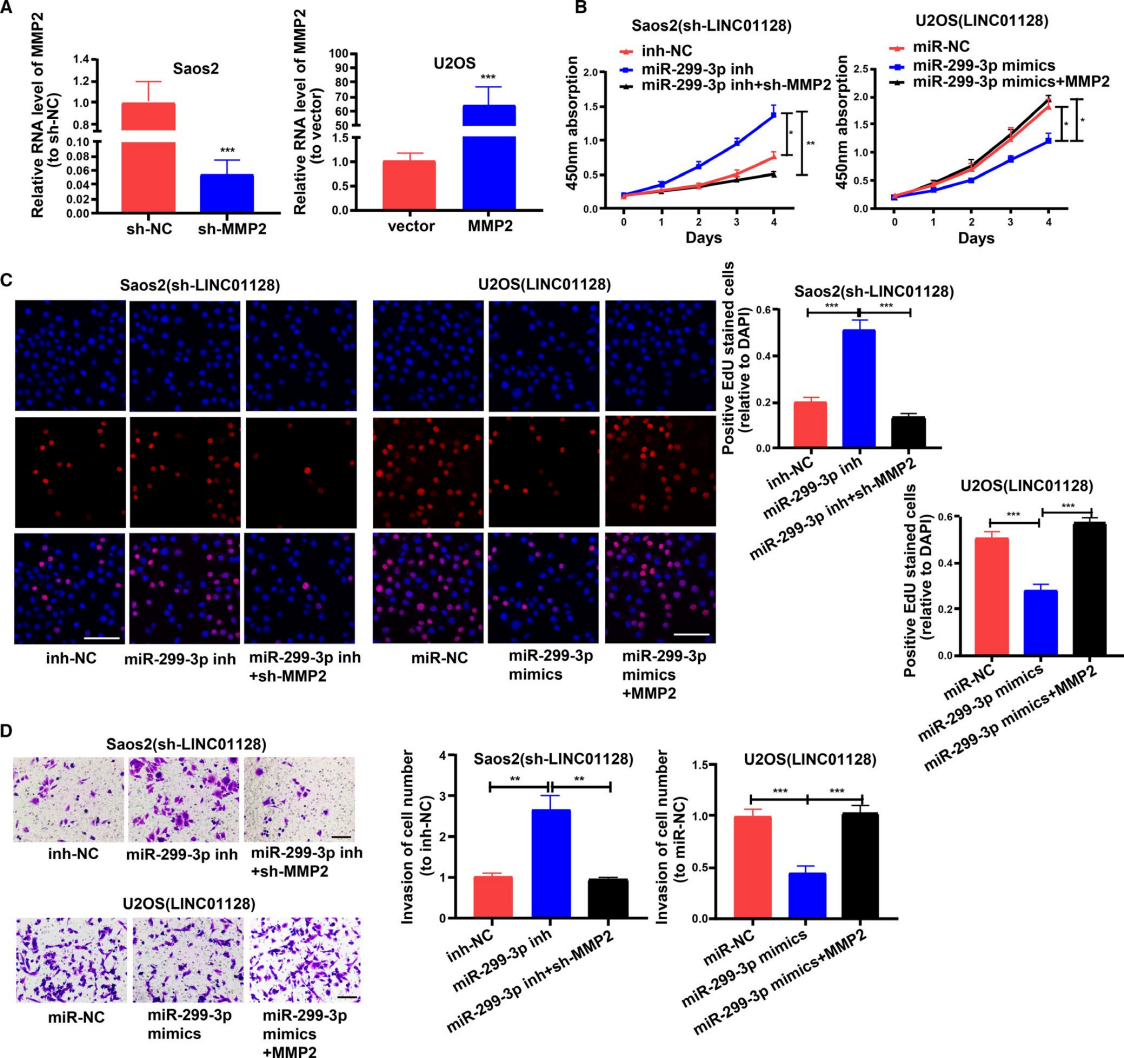
*LINC01128* affects proliferation and invasion of OS cells through collaborating with miR‐299‐3p and *MMP2*. (A) Transfection efficiency of sh‐*MMP2* and *MMP2* overexpression vector. (B, C) CCK‐8 and EdU assays showing proliferation of Saos2(sh‐*LINC01128*) and U2OS(*LINC01128*) cells after transfection (bar = 100 μm). (D) Transwell assay showing invasion of Saos2(sh‐*LINC01128*) and U2OS(*LINC01128*) cells after transfection (bar = 100 μm). ^*^ *P* < .05, ^**^ *P* < .01 and ^***^ *P* < .001

Saos2 and U2OS cells stably transfected with sh‐*LINC01128* or *LINC01128* overexpression vector were subjected to rescue experiments. The transfection efficiency of sh‐*MMP2* and *MMP2* are shown in Figure 7A. CCK8 and EdU assays were performed to analyse cell proliferation. Cell invasion was also measured. The results showed that the miR‐299‐3p inhibitor promoted cell proliferation and invasion of OS cells transfected with sh‐*LINC01128*, while sh‐*MMP2* reversed it (Figure 7B‐D). Further, miR‐299‐3p mimics inhibited cell proliferation and invasion of OS cells transfected with *LINC01128* overexpression vector, while *MMP2* overexpression vector reversed it (Figure 7B‐D).

### 3.7 LINC01128 and MMP2 jointly activate the Wnt/β‐catenin signalling pathway

**FIGURE 8:**
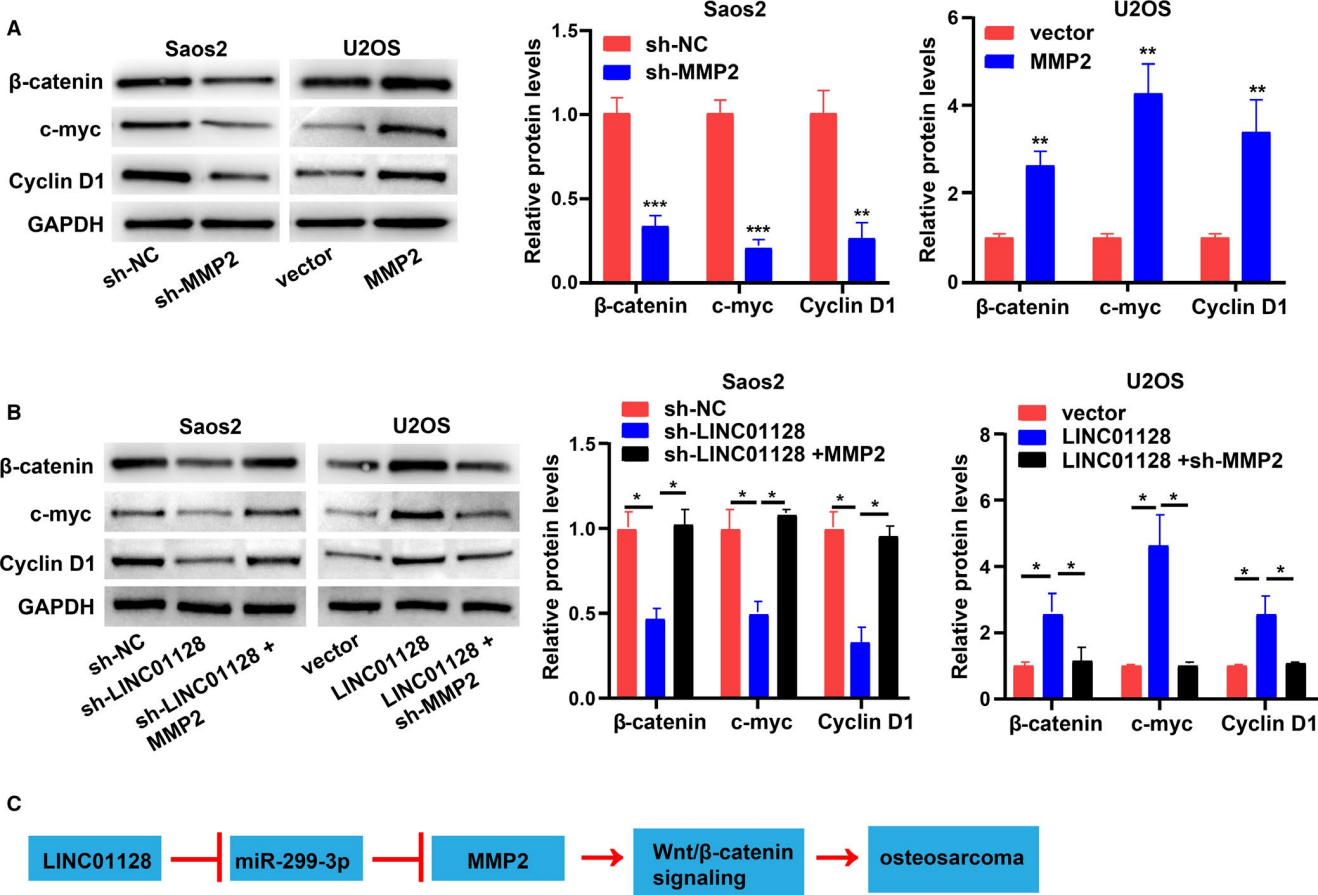
*LINC01128* and *MMP2* jointly activate the Wnt/β‐catenin signalling pathway. (A) Levels of Wnt/β‐catenin signalling pathway‐associated proteins in OS cells transfected with sh‐*MMP2* or *MMP2* overexpression vector. (B) Protein levels of Wnt/β‐catenin signalling pathway‐associated proteins in OS cells. (C) Proposed mechanism of *LINC01128* in OS *LINC01128* functions as a sponge of miR‐299‐3p, thus promoting MMP2 expression and activating the Wnt/β‐catenin signalling pathway, and finally facilitates OS. ^*^ *P* < .05, ^**^ *P* < .01 and ^***^ *P* < .001

A previous study demonstrated that OS progression is associated with the transcriptional activity of activated β‐catenin.^23^ Besides, some studies have also shown that *MMP2* is closely associated with the Wnt/β‐catenin signalling pathway.^24^, ^25^, ^26^ This study validates that *LINC01128* affects *MMP2* mRNA stability through competitive binding with miR‐299‐3p. Therefore, we investigated whether the Wnt/β‐catenin signalling pathway was activated by *LINC01128* through the positive regulation of *MMP2* in OS. The levels of the Wnt/β‐catenin signalling pathway‐associated proteins were measured using Western blotting in Saos2 and U2OS cells after transfection. The protein levels of β‐catenin, C‐myc and cyclin D1 were inhibited by sh‐*MMP2*, but promoted by overexpression of *MMP2* (Figure 8A), implying that *MMP2* may activate the Wnt/β‐catenin signalling pathway. Additionally, as shown in Figure 8B, sh‐*LINC01128* inhibited the protein levels of β‐catenin, C‐myc and cyclin D1, while, co‐transfection with *MMP2* overexpression vector reversed their expression. *LINC01128* overexpression vector promoted the protein levels of β‐catenin, C‐myc and cyclin D1, but the effects were reversed by co‐transfection with sh‐*MMP2* (Figure 8B). The above findings validate that *LINC01128* positively regulates *MMP2*, which activates the Wnt/β‐catenin signalling pathway (Figure 8C).

## 4 DISCUSSION

Recently, increasing number of studies have reported the pivotal roles of lncRNAs in the occurrence and development of OS.^27^, ^28^ Hu Y and his colleagues reported that LINC01128 expedites cervical cancer progression by regulating miR‐383‐5p/SFN axis.^21^ In the present study, we demonstrated that *LINC01128* is significantly overexpressed in OS tissues and cell lines, and promotes proliferation, migration and invasion of OS cells.

LncRNAs facilitate tumour development by sponging miRNAs and protecting their target mRNAs from repression.^29^, ^30^ The results of subcellular localization analysis suggest that *LINC01128* may play a role through competing endogenous RNA (ceRNA) mechanism. Bioinformatics analysis suggests that LINC01128 may bind with miR‐299‐3p. MiR‐299‐3p is a tumour suppressor gene, shown to inhibit the development of a variety of tumours, including cervical,^31^ thyroid^32^ and hepatocellular carcinoma^33^ among others. To explore the specific mechanism of LINC01128 in OS, we performed a series of experiments and demonstrated that *LINC01128* regulated the expression of *MMP2* by competitively binding to miR‐299‐3p, thereby regulating the pathophysiological process of OS.

Wnt/β‐catenin signalling is an evolutionarily conserved and versatile pathway. Several studies have implicated the Wnt/β‐catenin pathway in tissue homeostasis, embryonic development and various human diseases.^34^, ^35^, ^36^ Ectopic activation of this pathway leads to the accumulation of β‐catenin in the nucleus and stimulates the transcription of many oncogenes.^37^, ^38^ Previous studies have reported that abnormal activation of the Wnt signalling pathway plays an important role in OS pathogenesis. Therefore, in this study, we examined whether *LINC01128/* miR‐299‐3p/ *MMP2* had an effect on the Wnt/β‐catenin signalling pathway. Our results confirmed that *LINC01128*/miR‐299‐3p/*MMP2* functions *via* the Wnt/β‐catenin signalling pathway.

However, the study has several limitations. First, a larger tissue sample size of OS is required to further explore the clinical value of *LINC01128*. Second, whether there are other target genes or miRNAs which can interact with *LINC01128* requires further exploration.

In conclusion, *LINC01128* plays a carcinogenic role in OS *LINC01128* regulates the development of OS through sponging miR‐299‐3p to promote *MMP2* expression and activate the Wnt/β‐catenin signalling pathway. Our study is the first to identify *LINC01128* as a marker and prognostic target in OS. However, further in‐depth investigations are needed in the future prior to its potential clinical applications.

## CONFLICT OF INTEREST

The authors report no conflicts of interest.

## AUTHOR CONTRIBUTION

**Qiang Yao:** Data curation (lead); Methodology (lead); Writing‐original draft (supporting); Writing‐review & editing (supporting). **Ting Chen:** Conceptualization (lead); Writing‐original draft (lead); Writing‐review & editing (lead).

## ETHICAL APPROVAL AND INFORMED CONSENT

The present study was approved by the Ethics Committee of Shengjing Hospital of China Medical University. All population‐related studies were carried out according to the principles of good clinical practice and the World Medical Association Declaration of Helsinki. Written informed consent was signed by all the participants.

## Supporting information

Fig S1

Fig S2

Fig S3

Fig S4

Fig S5

Table S1

Table S2

Table S3

## Data Availability

The data pertaining to the current study are available from the corresponding author on reasonable request.
