# Supplementary material for: LINC01128 regulates the development of osteosarcoma by sponging miR‐299‐3p to mediate MMP2 expression and activating Wnt/β‐catenin signalling pathway
Source: J Cell Mol Med. 2020 Oct 27;24(24):14293–305. doi: 10.1111/jcmm.16046 (PMC7753992; doi:10.1111/jcmm.16046)
Supplement: Supplementary file 1 — Fig S1 [file JCMM-24-14293-s001.docx]

**Supplementary Figure 1**


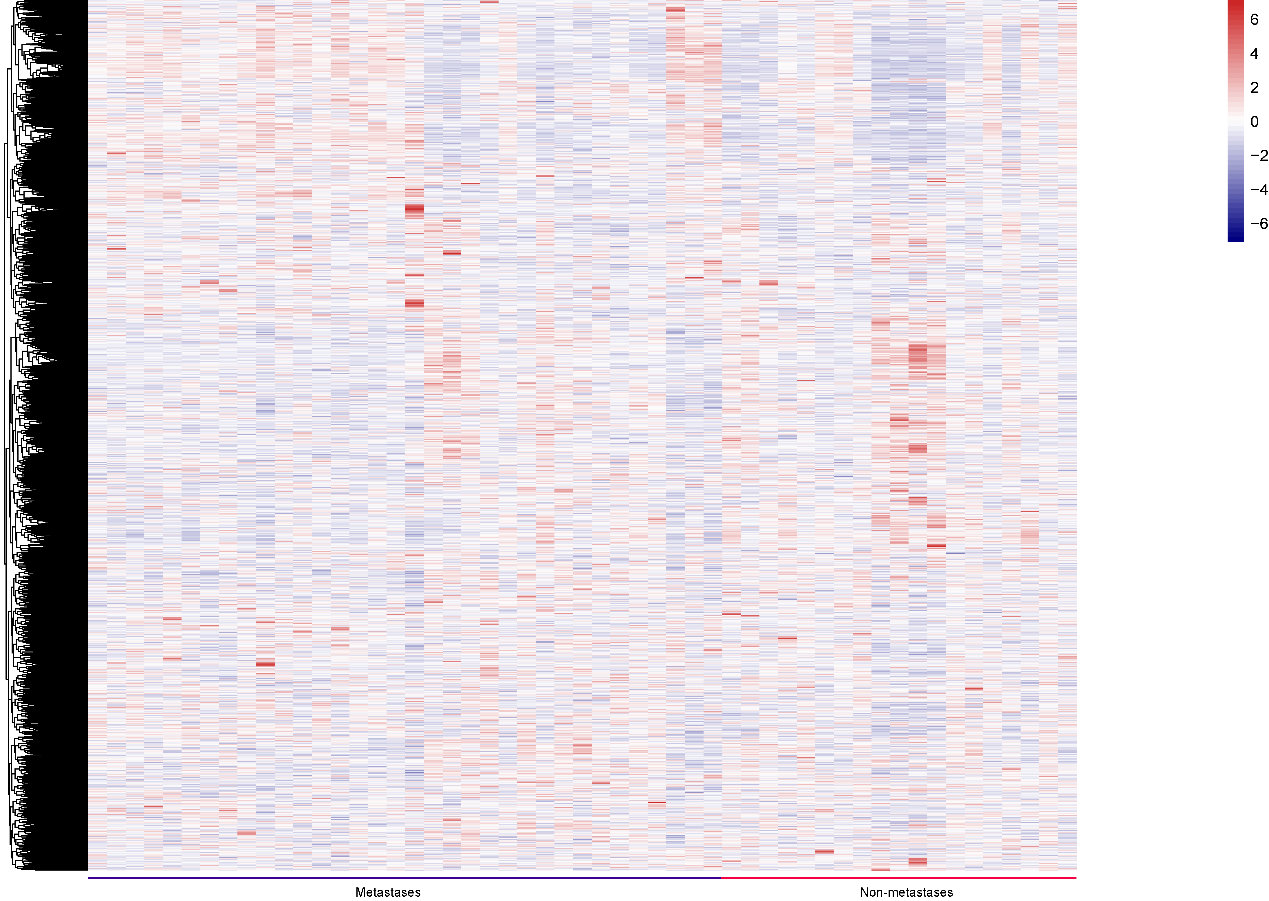


**Supplementary Figure 1.** Heatmap of **GSE21257** Microarray shows the expression profile of lncRNAs and mRNAs in OS samples. Pre-chemotherapy biopsies of osteosarcoma patients who developed metastases within 5yrs (n=34) were compared with pre-chemotherapy biopsies of osteosarcoma patients who did not develop metastases within 5yrs (n=19).
