# Supplementary material for: LINC01128 regulates the development of osteosarcoma by sponging miR‐299‐3p to mediate MMP2 expression and activating Wnt/β‐catenin signalling pathway
Source: J Cell Mol Med. 2020 Oct 27;24(24):14293–305. doi: 10.1111/jcmm.16046 (PMC7753992; doi:10.1111/jcmm.16046)
Supplement: Supplementary file 3 — Fig S3 [file JCMM-24-14293-s003.docx]

**Supplementary Figure 3**


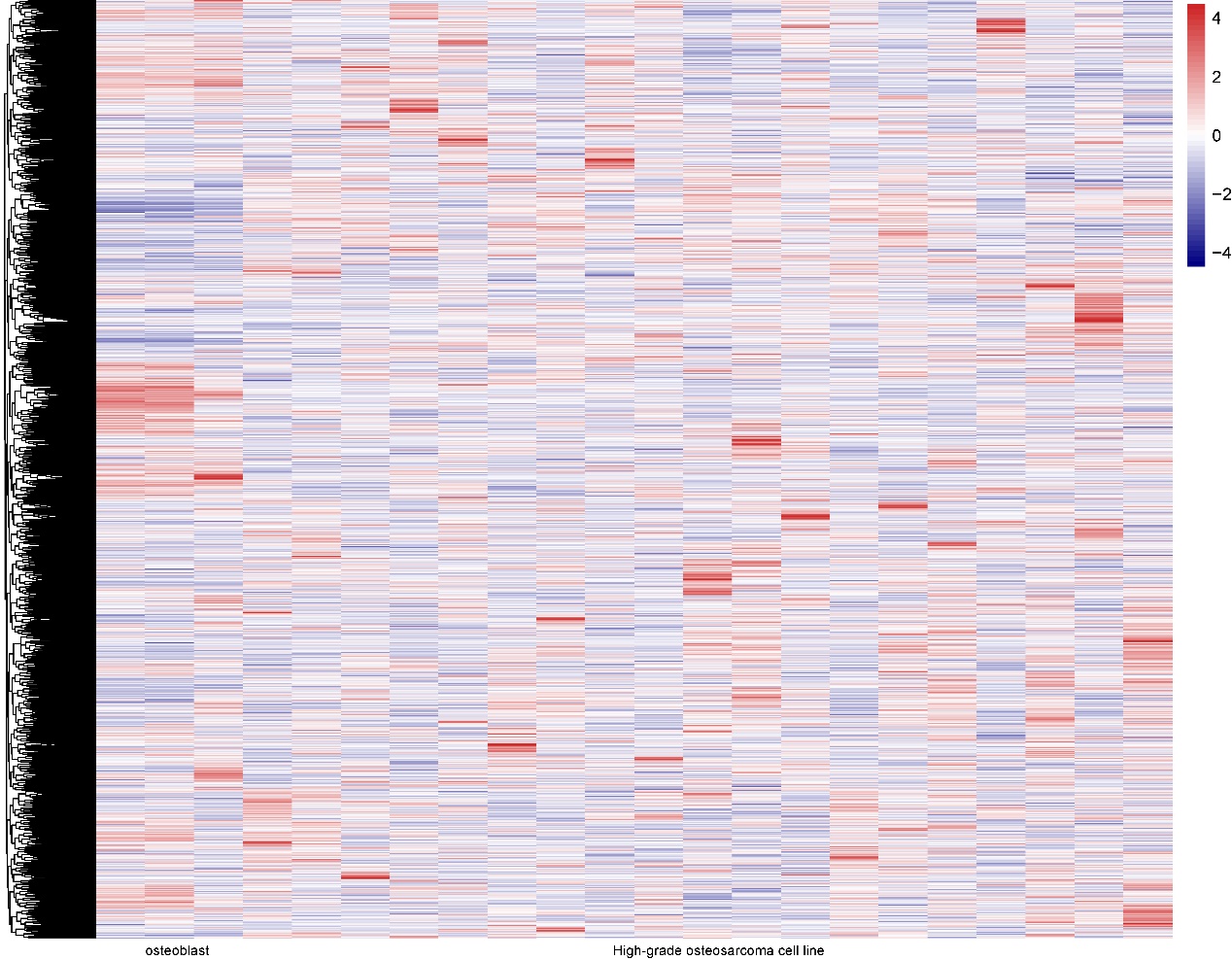


**Supplementary Figure 3.** Heatmap of **GSE42352** Microarray shows the expression profile of lncRNAs and mRNAs in OS samples. Comparison of gene expression patterns in 3 Osteoblasts和19 High-grade osteosarcoma cell lines.
