# Supplementary material for: LINC01128 regulates the development of osteosarcoma by sponging miR‐299‐3p to mediate MMP2 expression and activating Wnt/β‐catenin signalling pathway
Source: J Cell Mol Med. 2020 Oct 27;24(24):14293–305. doi: 10.1111/jcmm.16046 (PMC7753992; doi:10.1111/jcmm.16046)
Supplement: Supplementary file 4 — Fig S4 [file JCMM-24-14293-s004.docx]

**Figure S4**


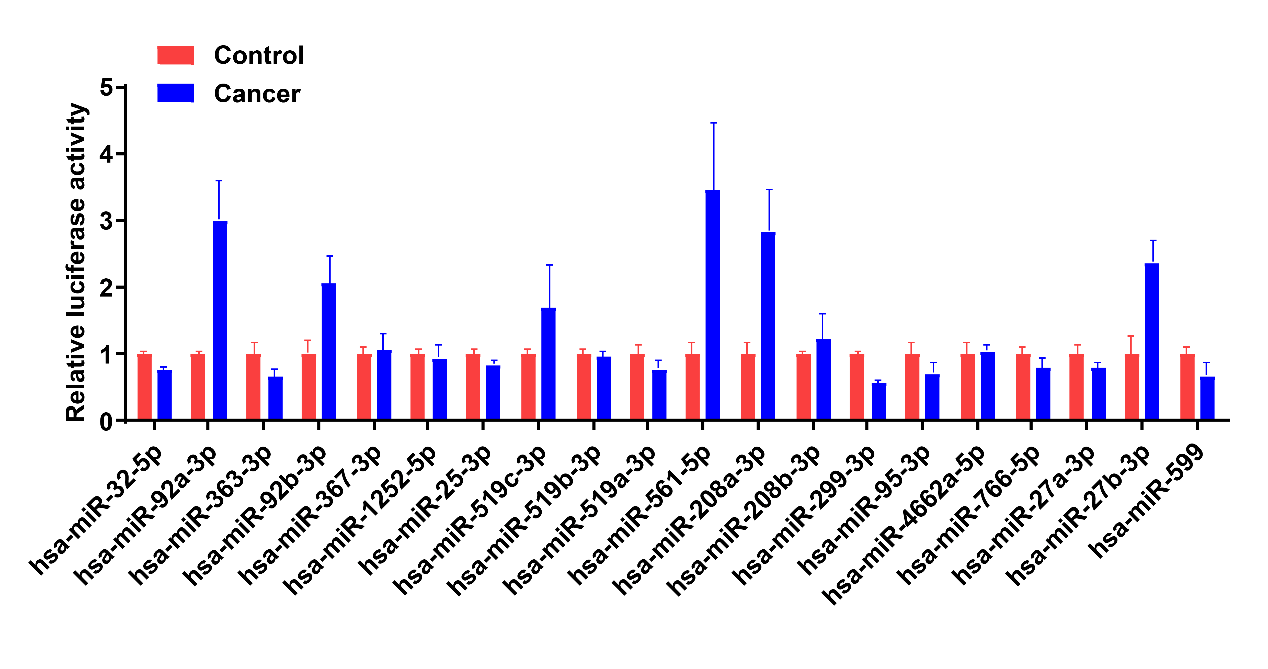


**Figure S4.** The expression of the 20 potential target miRNAs of *LINC01128* in OS samples. Among the 20 potential target miRNAs, miR-299-3p is the most obviously downregulated miRNA in OS tissues.
