# Supplementary material for: LINC01128 regulates the development of osteosarcoma by sponging miR‐299‐3p to mediate MMP2 expression and activating Wnt/β‐catenin signalling pathway
Source: J Cell Mol Med. 2020 Oct 27;24(24):14293–305. doi: 10.1111/jcmm.16046 (PMC7753992; doi:10.1111/jcmm.16046)
Supplement: Supplementary file 5 — Fig S5 [file JCMM-24-14293-s005.docx]

**Figure S5**


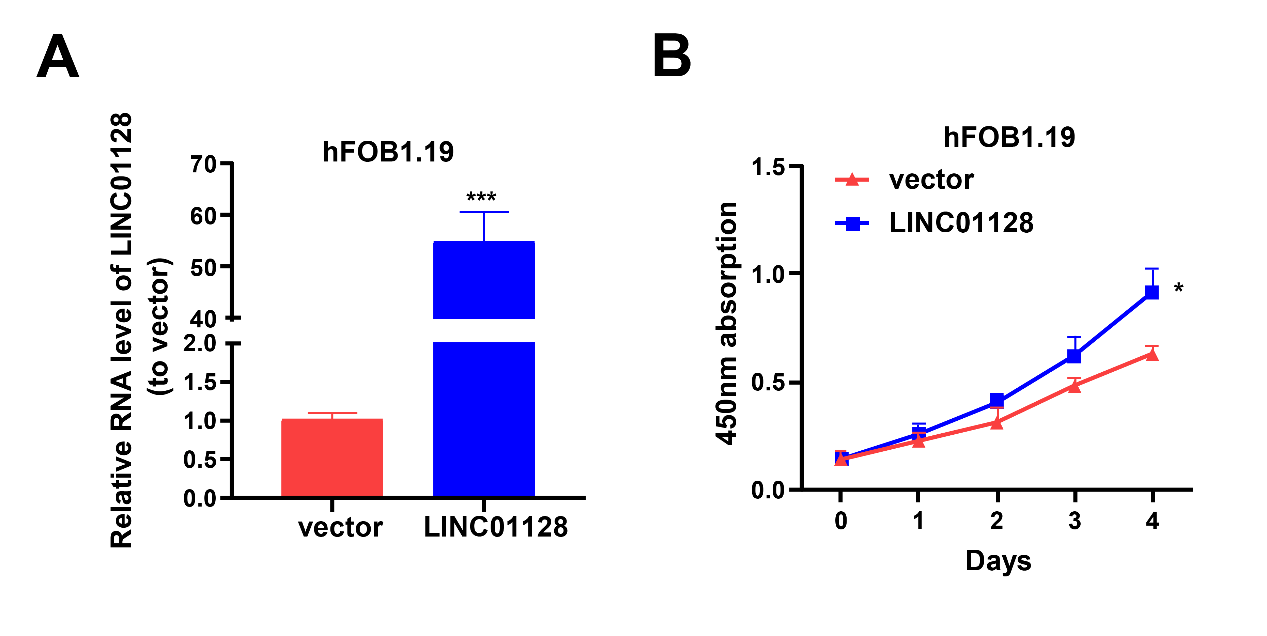


**Figure S5.** Effect of LINC01128 on the proliferation of hFOB1.19 cells. (A) Transfection efficiency of LINC01128 overexpression or negative control vector in hFOB1.19 cells. (B) CCK-8 assays confirmed the effect of LINC01128 overexpression on the proliferation of hFOB1.19 cells. ^*^*P* < 0.05 and ^***^*P* < 0.001.
