# Supplementary material for: LINC01128 regulates the development of osteosarcoma by sponging miR‐299‐3p to mediate MMP2 expression and activating Wnt/β‐catenin signalling pathway
Source: J Cell Mol Med. 2020 Oct 27;24(24):14293–305. doi: 10.1111/jcmm.16046 (PMC7753992; doi:10.1111/jcmm.16046)
Supplement: Supplementary file 6 — Table S1 [file JCMM-24-14293-s006.docx]

**Table S1.** **Sequences of primers for qRT-PCR and miRNA related sequence**

| **Name** |  | | **Sequence** |
| --- | --- | --- | --- |
| LINC01128 | | Forward | 5’- CAGAGGAGCTACGAAGGGAG -3’ |
|  |  | Reverse | 5’-CTGTGGAATCGCTTGGTACG -3’ |
| miR-299-3p | | Forward | 5'-ACACTCCAGCTGGGTTCGCCAAATGGTAGG-3' |
|  |  | Reverse | 5'-CTCAACTGGTGTCGTGGAGTCGGCAA TTCAGTTGAGATACACCC-3' |
| MMP2 | | Forward | 5'- TACAGGATCATTGGCTACACACC -3' |
|  |  | Reverse | 5'- GGTCACATCGCTCCAGACT 3' |
| GAPDH | | Forward | 5’- AGTAGAGGCAGGGATGATG -3’ |
|  |  | Reverse | 5’- TGGTATCGTGGAAGGACTC -3’ |
| U6 | | Forward | 5'- GGTCGGGCAGGAAAGAGGGC -3' |
|  |  | Reverse | 5'- CTAATCTTCTCTGTATCGTTCC -3' |
